# “We do it ourselves”: strengths and opportunities for improving the practice of harm reduction

**DOI:** 10.1186/s12954-023-00809-7

**Published:** 2023-06-09

**Authors:** Kasey Claborn, Jake Samora, Katie McCormick, Quanisha Whittfield, Frederic Courtois, Kyle Lozada, Daniel Sledge, Annie Burwell, Sandra Chavez, Jamie Bailey, Chris Bailey, Chelsea Dalton Pederson, Claire Zagorski, Lucas Hill, Fiona N. Conway, Lori Holleran Steiker, Jessica Cance, Jennifer Potter

**Affiliations:** 1 Steve Hicks School of Social Work, The University of Texas at Austin, Austin, TX USA; 2 Department of Psychiatry, Dell Medical School, The University of Texas at Austin, Austin, TX USA; 3 Addictions Research Institute, The University of Texas at Austin, Austin, TX USA; 4 The Center for Healthcare Services, San Antonio, TX USA; 5 Round Rock Fire Department Crisis Response Unit, Round Rock, TX USA; 6 ASHWell, Austin, TX USA; 7 Project Vida, El Paso, TX USA; 8 College of Pharmacy, The University of Texas at Austin, Austin, TX USA; 9 RTI International, Research Triangle Park, NC USA; 10 UTHSCA, San Antonio, TX USA

**Keywords:** Harm reduction, Drug use, Opioid, Qualitative methods, Stigma

## Abstract

**Background:**

Unprecedented increases in substance-related overdose fatalities have been observed in Texas and the U.S. since the onset of the COVID-19 pandemic and have made clear there is considerable need to reduce harms associated with drug use. At the federal level, initiatives have called for widespread dissemination and implementation of evidence-based harm reduction practices to reduce overdose deaths. Implementation of harm reduction strategies is challenging in Texas. There is a paucity of literature on understanding current harm reduction practices in Texas. As such, this qualitative study aims to understand harm reduction practices among people who use drugs (PWUD), harm reductionists, and emergency responders across four counties in Texas. This work would inform future efforts to scale and spread harm reduction in Texas.

**Methods:**

Semi-structured qualitative interviews were conducted with *N* = 69 key stakeholders (25 harm reductionists; 24 PWUD; 20 emergency responders). Interviews were transcribed verbatim, coded for emergent themes, and analyzed using Applied Thematic Analysis with Nvivo 12. A community advisory board defined the research questions, reviewed the emergent themes, and assisted with interpretation of the data.

**Results:**

Emergent themes highlighted barriers to harm reduction at micro and macro levels, from the individual experience of PWUD and harm reductionists to systemic issues in healthcare and the emergency medical response system. Specifically, (1) Texas has existing strengths in overdose prevention and response efforts on which to build, (2) PWUD are fearful of interacting with healthcare and 911 systems, (3) harm reductionists are in increasing need of support for reaching all PWUD communities, and (4) state-level policies may hinder widespread implementation and adoption of evidence-based harm reduction practices.

**Conclusions:**

Perspectives from harm reduction stakeholders highlighted existing strengths, avenues for improvement, and specific barriers that currently exist to harm reduction practices in Texas.

## Introduction

Harm reduction and overdose prevention efforts are increasingly prioritized to address the opioid crisis which has reached historic highs of almost 108,000 overdose fatalities during 2021 [1]. This has resulted in increased federal funding and new policy models focused on harm reduction to improve access to naloxone, fentanyl testing strips [2], and syringe service programs [3]. Harm reduction interventions promote safer drug use practices and have strong empirical support for reducing drug overdose fatalities and facilitating changes in harmful drug use behaviors on a wide scale [4–6]. It is important to understand how diverse political and cultural landscapes influence or impede the adoption and effective uptake of the United States’ national harm reduction strategy at a regional and local level.

Each state in the United States has a unique landscape and has varied approaches to providing services in harm reduction, prevention, and treatment. Texas faces several challenges in combating the worsening overdose crisis. The expansive geography of the state creates barriers to widespread dissemination and implementation of state-level initiatives, requiring significant economic resources and staffing to support equitable implementation and sustainability across rural, urban, and tribal communities. Further, improved collection and aggregation of harm reduction-related data (fatal/non-fatal overdose incidence, naloxone administration) is needed to drive prevention efforts and resource allocation [7]. Additionally, Texas legislation impedes the implementation and adoption of some harm reduction services [8]. For example, Texas is one of 11 states in which syringe service programs are illegal [9] and drug paraphernalia law prohibits the possession and distribution of fentanyl testing strips [10]. “As of May 2023, there has not been any modification by the Texas Legislature to either expand or discontinue the Bexar County pilot program and thus, without reliable infrastructure to attest of its effectiveness. Without clarity on policy emanating from the Texas Legislature, there are very few local District Attorneys’ offices that have been willing to support local harm reduction efforts by even de-criminalizing possession of paraphernalia. The lack of reliable, free, and centralized database makes it even more difficult to integrate and coordinate for greater efficacy of engagement with at-risk populations. Current harm reduction methods are confined to traditional outreach methods and include prevention kits (smoke and hygiene kits) and distribution of naloxone”.

The Texas Targeted Opioid Response (TTOR) program, a public health initiative operated by the Texas Health and Human Services Commission, was established to respond to some of these challenges by expanding access to prevention, early intervention, treatment, and recovery support services (https://txopioidresponse.org). TTOR has facilitated the statewide distribution of naloxone and training of healthcare professionals and community members to effectively respond to suspected opioid overdoses (www.TXOTI.org). TTOR also funds a variety of projects to enhance overdose surveillance and improve services for PWUD, such as TxCOPE (www.txcope.org) and Be Well, Texas (www.bewelltexas.org). The TTOR program has made valuable and important advances towards improving opioid-related treatment and prevention services across the state.

Existing literature on harm reduction in Texas is severely limited. Much of the existing research focuses on reducing alcohol-related harms among youth and college-aged populations [11–14]. There have been growing efforts to understand harm reduction in relation to opioid use in Texas including use of syndromic surveillance to identify hot spots [15, 16] and harm reduction education among student pharmacists [17, 18]. Data demonstrate a worsening overdose crisis in Texas. In 2020, the State of Texas recorded over 4000 drug overdose deaths [19], 4000 opioid-related poison center calls [20], and nearly 8000 opioid-related emergency room visits [21]. These rapid increases have prompted some counties to declare an overdose public health emergency [22, 23]. A 2021 qualitative study among people who use opioids receiving services at mobile harm reduction outreach sites in Austin found that most participants reported the presence of fentanyl in heroin and other drugs they consumed [24]. The authors identified a need for expanded harm reduction service delivery specifically focused on fentanyl and other potent synthetic opioids.

Taken together, an understanding of current harm reduction efforts across the varied regions in Texas is needed to identify existing strengths, current gaps, and opportunities to advance efforts to improve statewide overdose prevention and response efforts. This qualitative study used community-engaged research methods to examine the perspectives of PWUD, harm reductionists, and emergency responders in Texas to better understand barriers to practicing harm reduction and providing evidence-based overdose prevention services. Specifically, we sought to answer the following research questions: (1) what barriers exist for PWUD to engage with the healthcare system following an overdose? (2) what strengths and challenges exist for harm reduction organizations in serving their clients in Texas? and (3) what are harm reductionists’ perspectives on how policy influences harm reduction philosophy and practice in Texas?

## Methods

This secondary analysis of qualitative data from a larger parent study [7] was collaboratively conceptualized by community harm reductionists, people with lived experience including active drug use, and overdose prevention researchers working together to improve harm reduction efforts across Texas. All study procedures were approved by The University of Texas at Austin institutional review board.

**Fig. 1:**
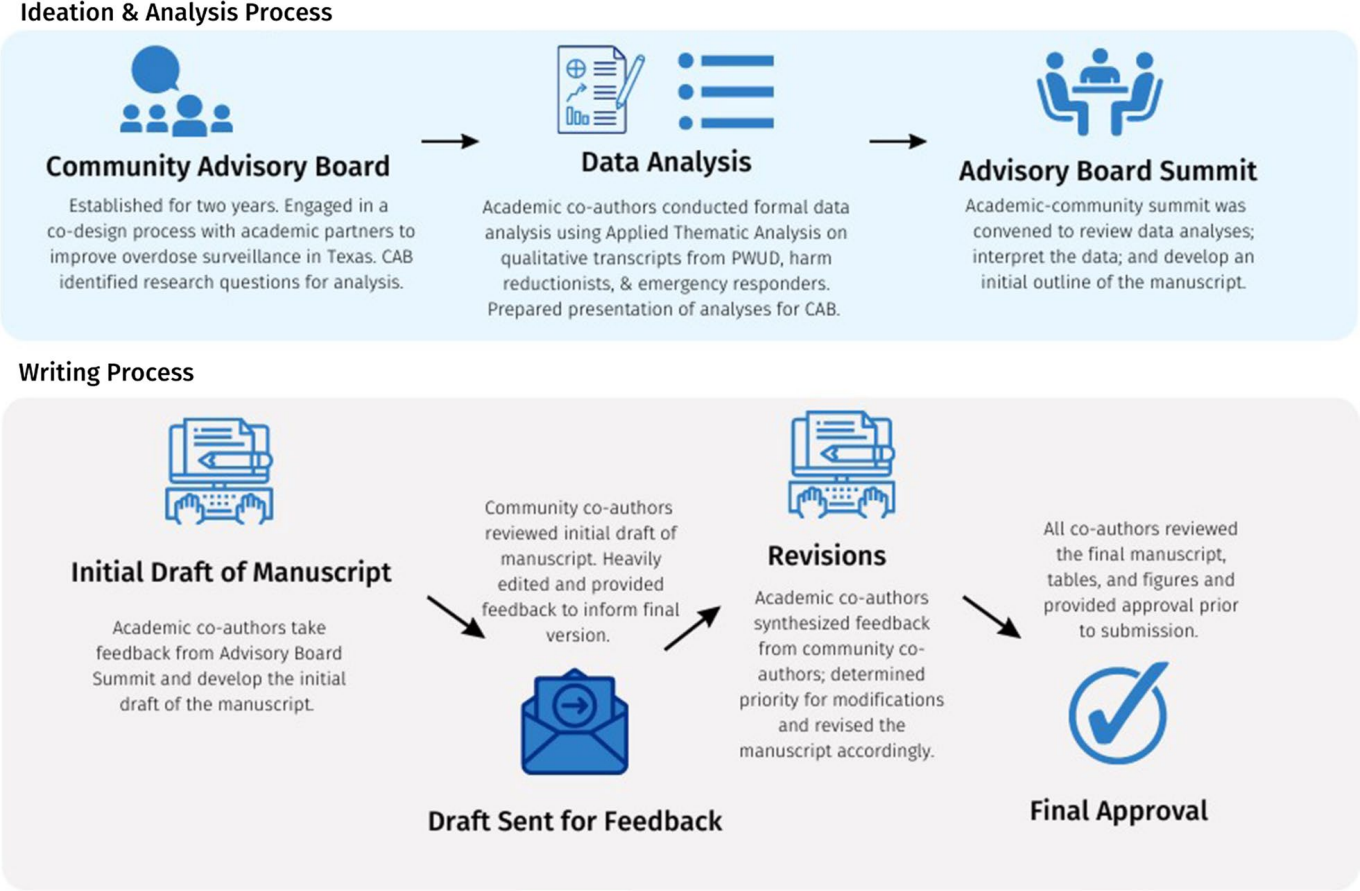
Collaborative manuscript development process

*Conceptual Framework.* We used community-based participatory research (CBPR) methods to elevate the perspectives, experiences, and needs of PWUD, harm reduction workers, and emergency responders in a co-collaborative effort. See [16] for a detailed description. Our community-academic partnership was fostered through regional community advisory boards (CABs) located in four urban Texas counties: Bexar (San Antonio), El Paso, Travis (Austin), and Williamson (Round Rock/Georgetown), which brought together key stakeholders in harm reduction to co-design and implement a community-based overdose reporting platform, TxCOPE, or Texans Connecting Overdose Prevention Efforts. Our collaborative development process for this manuscript can be viewed in Fig. 1. CAB members participated in developing the interview guide and research questions, recruiting participants, and assisted with interpretation of study results.

*Qualitative Interviews with Key Stakeholders.* Qualitative interviews were conducted virtually and in-person among a series of *N* = 69 key stakeholders (*n* = 24 PWUD; *n* = 25 harm reductionists; *n* = 20 emergency responders) across Bexar, El Paso, Travis, and Williamson Counties in Texas. These interviews lasted 60–90 min and were audio recorded. Participants received a $30 incentive for their time. Semi-structured interview and debriefing guides were created for each participant group. The interview guides were composed of a combination of structured, open-ended questions, and follow-up probes, which provided flexibility for interviewers to adapt and clarify questions as needed.

### Participants

*Eligibility.* The inclusion criteria for PWUD included: 1) 18 years or older; 2) non-prescribed use of an opioid or stimulant in the past three months, 3) Texas resident, and 4) fluent in English. The inclusion criteria for harm reductionists and emergency responders included: 1) 18 years or older, 2) employed at an overdose prevention/harm reduction organization or at an EMS or Fire Department in one of the target counties, and 3) fluent in English. Exclusion criteria included the inability or unwillingness to provide consent and being actively suicidal or psychotic.

*Recruitment.* Recruitment efforts included snowball sampling methods such as in-person communications, flyers, e-mails, reaching out to known contacts by telephone, and word of mouth through CABs. Prospective participants were screened using a short survey conducted over the phone or through email. If the inclusion criteria were met, the research team obtained verbal informed consent.

### Data collection

Two trained research staff members conducted qualitative interviews using a videoconference platform or face-to-face. One researcher directed the interview and the other served as a notetaker. Once the interview concluded, the audio recordings were transcribed verbatim, de-identified and cleaned.

### Data analysis

We used Applied Thematic Analysis to guide our data analysis [25]. Emergent themes were identified based on the a priori research questions. A working codebook and framework matrix were generated based on the themes for each participant strata (PWUD, harm reductionists, first responders). Framework matrix is a systematic method of categorizing and organizing qualitative data into a matrix output: rows (cases), columns (codes), and cells of summarized data which provide a structure to systematically deduce the data in order to analyze it by case and by code [26]. The transcripts were double-coded by two trained research assistants. Discrepancies were resolved through discussion with a third coder. The master coded transcripts were entered into NVivo12. Code reports were generated and followed by an inductive phase using analytic matrix display to summarize emerging themes. Data across the three strata were triangulated.

## Results

### Sample characteristics

Our final sample yielded *N* = 69 participants, with harm reductionists representing the largest subsample (*n* = 25), followed by PWUD (*n* = 24), and emergency responders (*n* = 20). The majority of the sample were men (58%), white (78%), and non-Hispanic/Latino (61%). Most harm reductionists were women (52%) and had obtained a graduate or professional degree (44%), while PWUD and emergency responders who were mostly men (54% and 85%, respectively) and completed some college or a 2-year degree (50% for both groups). In terms of age, most harm reductionists were slightly younger (ages 25–34; 36%) and PWUD were slightly older (ages 35–44; 46%) while emergency responders varied across age groups. Almost half (45%) of emergency responders identified as Christians, while most of both PWUD and harm reductionists ascribed to another religion not listed (58% and 32%, respectively). Income level varied across subsamples, with PWUD earning the least (less than $25,000/year), emergency responders earning the most ($50–$74,999/year), and harm reductionists earning in between ($25–49,999/year). See Table 1 for additional participant characteristics.

**Table 1:** Participant characteristics (*N* = 64)

	People who use drugs (*n* = 24)	Emergency responders (*n* = 20)	Harm reductionists (*n* = 25)	Total (*n* = 69)
	*N* (%)	*N* (%)	*N* (%)	*N* (%)
*Age*				
18–24	4 (16.6)	1 (5.0)	1 (4.0)	6 (8.7)
25–34	4 (16.6)	6 (30.0)	9 (36.0)	19 (27.5)
35–44	11 (45.8)	6 (30.0)	7 (28.0)	24 (34.8)
45–54	2 (8.3)	6 (30.0)	4 (16.0)	12 (17.4)
55+	3 (12.5)	1 (5.0)	4 (16.0)	8 (11.6)
*Sex at birth*				
Male	13 (54.2)	18 (90.0)	11 (44.0)	42 (60.9)
Female	11 (45.8)	2 (10.0)	14 (56.0)	27 (39.1)
*Gender identity*				
Man	13 (54.2)	17 (85.0)	10 (40.0)	40 (58.0)
Woman	11 (45.8)	2 (10.0)	13 (52.0)	26 (37.7)
Genderqueer	0 (0.0)	1 (5.0)	2 (8.0)	3 (4.3)
*Race*				
African American or Black	1 (4.2)	0 (0.0)	3 (12.0)	4 (5.8)
Asian	0 (0.0)	2 (10.0)	2 (8.0)	4 (5.8)
White/Caucasian	19 (79.2)	17 (85.0)	17 (68.0)	53 (77.8)
American Indian or Alaska Native	0 (0.0)	0 (0)	1 (4.0)	1 (1.5)
Other	6 (23.0)	1 (5.0)	1 (4.0)	8 (11.6)
Prefer not to answer	0 (0.0)	0 (0.0)	1 (4.0)	1 (1.5)
*Ethnicity*				
Hispanic or Latino	10 (41.7)	3 (15.0)	11 (44.0)	24 (34.8)
Non-Hispanic or Latino	13 (54.2)	16 (80.0)	13 (52.0)	42 (60.9)
Other	1 (4.2)	1 (5.0)	1 (4.0)	3 (4.3)
*Religion*				
Christian	6 (25.0)	9 (45.0)	8 (32.0)	23 (33.3)
Buddhist	1 (4.2)	0 (0.0)	1 (4.0)	2 (2.9)
Jewish	1 (4.2)	0 (0.0)	0 (0.0)	1 (1.5)
Atheist	2 (8.3)	6 (30.0)	7 (28.0)	15 (21.7)
Other	14 (58.3)	5 (25.0)	8 (32.0)	27 (39.1)
N/A	0 (0.0)	0 (0.0)	2 (8.0)	2 (2.9)
*Education level*				
Some grade school	1 (4.2)	0 (0.0)	0 (0.0)	1 (1.5)
Some high school	1 (4.2)	0 (0.0)	0 (0.0)	1 (1.5)
High school diploma or GED	8 (33.3)	0 (0.0)	2 (8.0)	10 (15.5)
Some college or 2-year degree	12 (50.0)	10 (50.0)	4 (16.0)	26 (37.7)
4-year college graduate	1 (4.2)	9 (45.0)	8 (32.0)	18 (26.1)
Some school beyond college	1 (4.2)	0 (0.0)	0 (0.0)	1 (1.5)
Graduate or professional degree	0 (0.0)	1 (5.0)	11 (44.0)	12 (17.4)
*Income*				
Less than $25,000	11 (45.8)	0 (0.0)	4 (16.0)	15 (21.7)
$25,000–$49,999	7 (29.2)	1 (5.0)	14 (56.0)	22 (31.9)
$50,000–$74,999	4 (16.7)	7 (35.0)	2 (8.0)	13 (18.8)
$75,000–$99,999	1 (4.2)	6 (30.0)	3 (12.0)	10 (15.5)
Over $100,000	0 (0.0)	6 (30.0)	0 (0.0)	6 (8.7)
Don’t know/prefer not to answer	1 (4.2)	0 (0.0)	2 (8.0)	3 (4.4)
*Role in overdose reporting*				
Emergency department/hospital employee	N/A	4 (16.6)	0 (0.0)	4 (5.8)
EMS	N/A	12 (50.0)	0 (0.0)	12 (17.4)
Fire department	N/A	6 (25.0)	0 (0.0)	6 (8.7)
Harm reductionist	N/A	1 (4.1)	19 (76.0)	20 (29.0)
Law enforcement officer	N/A	1 (4.1)	0 (0.0)	1 (1.5)
Substance use treatment provider	N/A	0 (0.0)	2 (8.0)	2 (2.9)
N/A	0 (0.0)	0 (0.0)	4 (16.0)	4 (5.8)

### Emergent themes for barriers to engaging in harm reduction in Texas

Emergent themes highlighted strengths and obstacles to engaging in evidence-based harm reduction practices in Texas across the healthcare, carceral, legislative, and harm reduction systems. Identified barriers are described below according to each system. Importantly, participants emphasized the strong community bond and resilience among PWUD and harm reduction organizations in Texas in light of the barriers and challenges collectively experienced. See Table 2 for representative quotes by theme.

**Table 2:** Emergent themes for barriers to harm reduction in Texas

Topic	Emergent theme	Representative quotes	Endorsement counts^+^
*Healthcare not Reducing Harm for PWUD*	*Stigmatizing behavior by healthcare providers highlight negative experiences of PWUD:* ERs noted stigmatizing behavior by first responders and emergency dept personnel HRs noted experiencing stigma by emergency dept personnel, physicians, and SUD treatment providers PWUD noted experiencing stigma by emergency dept personnel	*"…it’s always fascinatin’ to me when we were doin’ outreach and bring someone in with a compound fracture, and doctors thought they did that shit on purpose so they could get drugs from the emergency department. And it’s just crazy. But it perpetuates the whole stigma…” (108, Harm Reductionist)* *"So when I went to the hospital…and I went in there on a wheelchair I couldn’t even walk. He said, ‘Uh, we’ll give you a couple muscle relaxers. We’ll give you something for the pain, and you’ll be okay. Go away.’ Well two weeks later, it got to the point where I couldn’t even hold my bowels. It was real bad. So I went back to the same hospital…and a different doctor came…he said, ‘Do you have an attorney?…Because, uh, they should’ve never let you leave from this hospital two weeks prior’” (160, PWUD)* *"There's a huge stigma. And then—but they're taking away of, like, you know, these are people, these are someone's son or daughter…there's people that just don't care. Like, if somebody overdoses, they're like, ‘Well, you know, they made that choice.’”(150, Emergency Responder)*	*n* = 11 HRs *n* = 14 PWUD *n* = 3 ERs
*PWUD will not Engage in 911 System after an Overdose*	*PWUD will not call 911 due to fear of being arrested and incarcerated* ERs, HRs, and PWUD all indicated that PWUD will not call 911 (emergency management services) due to fear of legal repercussions; if they do call 911, they won’t share info related to OD	*“…at the time, I’d be worried about them, like wantin’ to search my place-or-um, you know, or the person dies. You know I’d be worried about them coming back and trying to put it on me, you know” (135, PWUD)* *“I would also be hesitant to call 911 for fear of, uh, and it sounds so selfish, given the fact that a life is on the line, but I would als—I’d be fearful that if I was using that the emergency responders and police would charge me with something” (123, PWUD)*	*n* = 10 HRs *n* = 18 PWUD *n* = 9 ERs
*Harm Reduction Organizations not Reaching Entire Community of PWUD*	*Minority groups are likely not being reached by harm reduction organizations; lack of equitable distribution of services* FRs noted certain subpopulations of PWUD will not call 911/EMS for overdose due to EMS’ perception that certain groups (i.e. unhoused) don’t want help or have naloxone at home HRs also noted certain subgroups of the unhoused populations (i.e., veterans, groups living further in the woods), gender minorities, racial ethnic minorities (i.e., Black, Native American), and rural areas aren’t being served sufficiently PWUD also noted certain subgroups of the unhoused population aren’t being served sufficiently	*"But we can pretty much assume that the vast majority of people who meet the criteria for receiving these services and who could benefit from interaction around drug poisoning risk, around drug poisoning prevalence are just outside of the whole milieu altogether” (102, Harm Reductionist)* *"I mean people of color are wildly, um, you know, underrepresented in the population we serve. Uh, we see a disproportionately large number of men. I think the sex work community could benefit from our services and don’t…while we’ve reached into certain communities, there’s still some that we haven’t quite touched” (103, Harm Reductionist)*	*n* = 13 HRs *n* = 3 PWUD *n* = 3 ERs
*“Abstinence-Only” Philosophy Prevails over Harm Reduction in Texas*	*Harm reduction practices are neglected in “abstinence-only” models and collaboration between harm reduction organizations and healthcare providers are scarce* FRs noted that general knowledge and willingness to promote harm reduction programs embedded in EMS is not present in healthcare settings, with those settings more abstinence-based; lack of ability for EMS to follow-up with patient interaction when transferred to ED HRs noted prevalence of abstinence-only philosophy in SUD recovery and treatment settings leads to a neglection of harm reduction practice, which leads to ineffective outcomes for PWUD seeking services and potentially increases risk of mortality for PWUD (i.e. not talking about naloxone in these settings); partnerships with testing services, wound care services, MAT clinics, EMS providers are scarce PWUD noted abstinence-only philosophy in SUD treatment, recovery, and otherwise behavioral health settings and the potential consequences of sharing substance use/OD info to these professionals leads to PWUD not disclosing information in these settings; not receiving medication for pain	*"So in addiction treatment as a whole, the industry itself is implicated in denying people treatment, discharging people from treatment, and mistreating people for continuing to use substances” (102, Harm Reductionist)* *“…we were collaborating with [Local HIV Prevention Program]. But it was a struggle. It was a huge struggle. There was institutionalized stigma. You know, our clients were being treated poorly, and so it was just horrific until I got fed up and decided to just, you know, move offices…” (107, Harm Reductionist)* *"…this abstinence-based-only mentality that, um—that a lot of people here have, um, you know, permeates out into the larger community. And, um, you know, as unsuccessful as that has been at treatin' people with, uh, this disorder, it doesn't matter. It, um—it's what they continue to go with” (108, Harm Reductionist)*	*n* = 7 HRs *n* = 6 PWUD *n* = 3 ERs
*Funding for Harm Reduction in Texas as Inadequate*	*Harm reduction organizations are not adequately funded to serve PWUD communities nor keep relevant information on them* FRs highlighted insufficient funding of behavioral health and EMS services generally in Texas HRs noted inadequate funding for operations with regard to staff, frequency of outreach, and capacity to collect data related to overdose PWUD expressed concerns that certain programs have unstable funding to deliver harm reduction services	*"So we've hit a funding issue. Um, and it sounds like we might end up getting subsumed into THRA (Texas Harm Reduction Alliance). It's uncertain. Um, so, uh, I feel like if we're not well staffed, then, yeah, things—it's just a matter of kind of survival at that point 'cause we have so many people to see” (101, Harm Reductionist)* *"I feel that um, what needs to be done is we need more funding, so that we can be out there longer. Um, so that we can create a um—we just need to be out there longer. We need to be doing the work longer…” (116, Harm Reductionist)* *“I think that, uh, you know, to—funding for someone to report all that information would be key. You know? So that its accurate, we have one person or, you know? Because data, you know, data is really important.” (109, Harm Reductionist)*	*n* = 9 HRs *n* = 6 PWUD *n* = 3 ERs
*Absence of a Good Samaritan Law in Texas*	*Lack of a Good Samaritan Law (GSL) is a huge deterrent for calling 911 during an overdose* FRs noted lack of a GSL discourages PWUD for calling for help during an overdose HRs also noted lack of a GSL discourages PWUD for calling for help during an overdose, creates gaps in overdose information and the perception that leadership at the state level does not want to reduce overdose fatalities PWUD expressed uncertainty around the GSL and stated the current protections discourages PWUD from seeking care during an overdose; leads to incarcerations and PWUD not receiving necessary care during OD	*" the war on drugs is like, oh, you have drugs or you-you have a, you know, a diagnosis, I’m going to put you in jail for it. You know, um, so a-and the fact that we don’t have amnesty or-or the good Samaritan law, you know, um— That’s a big impact. " (146, Emergency Responder)* *“ Uh, but the bottom line is, like, you know, with or without a good Samaritan law, some people are gonna call 911, some people are just not, no matter what” (112, Harm Reductionist)* *“I feel like if people knew that they weren’t going to get in trouble for calling 9-1-1 and having, you know, EMS come and pick somebody up or dropping somebody off at the hospital or whatever… I think it’d be different… the Good Samaritan law is…not real” (129, PWUD)*	*n* = *6* HRs *n* = 4 PWUD *n* = 1 ERs
*Paraphernalia Law limits Harm Reduction Organizations from Offering Empirically-supported Services*	*Fentanyl testing strips and syringe service programs (SSPs) are not offered by harm reduction organizations* HRs noted SSP are not legal and prevents service provision (i.e., not being able to use federal grant funding for SSP); concerns that clients are concerned about fear of accessing their services, spreading of HIV/HCV, clients reusing needles, and challenges around advocating for SSP through collecting information from clients PWUD perceived that SSPs are illegal which leads to clients being hesitant or resistant to accessing HR outreach services; difficult to obtain clean syringes; means for drug testing not accessible	*"Like, we have people who come to us from Killeen, um, and they say that, you know, if they ever get stopped and the police see syringes, even if they're unopened they destroy them. If they see Narcan, they take it and confiscate it. So this happens a lot." (101, Harm Reductionist)* *"…syringe exchange is not legal in Texas, and that’s really um stopping us from meeting then what we need to do—the people that we need to meet and the demand that we need to meet. We are very—we’re-we’re not doing it, we’re just not—we’re not doin’ it.” (116, Harm Reductionist)*	*n* = 10 HRs *n* = 4 PWUD *n* = 0 ERs

^+^The overall study sample totaled *N* = 69, including *n* = 25 Harm Reductionists (HRs), *n* = 24 People Who Use Drugs (PWUD), and *n* = 20 Emergency Responders (ERs)

#### Perceived strengths to current harm reduction efforts in Texas

##### Harm reduction organizations combine efforts to advance harm reduction philosophy and practice in Texas

Participants described existing harm reduction efforts in Texas as having several strengths from which to build on. The following specific themes emerged: (1) the passion and commitment Texas harm reductionists have for their work and the population served; (2) possessing a strong person-centered approach; (3) an established statewide network of harm reduction groups across Texas; (4) harm reduction organizations have trust of PWUD in the community and engage with gatekeepers; (5) harm reduction organizations collaborate with local organizations including the local mental health authority, faith-based organizations, and community-wide boards; and (6) advocating for a Texas drug users’ union. These themes are exemplified by a harm reductionist who stated: “I think current harm reduction efforts in Texas are very strong due to the passion that harm reductionists have for the work. Many of these people have lived experience with SUD [substance use disorder] and have a strong person-centered approach in the work they do and a commitment to the work that shows in their services delivery. Also, there is a strong statewide network of harm reduction groups across Texas that meet regularly to troubleshoot challenges and barriers encountered. For example, the Texas Harm Reduction Alliance recently hosted a meeting of different harm reduction groups across the state to discuss Narcan shortages. They have also previously hosted a legislative workgroup where harm reduction groups across the state came together to work on the passing of bills to promote the work we do such as safe syringe programs” (140, HR).

##### PWUD provide care and support for each other

Both PWUD and harm reductionists emphasized the importance of community and preserving trust between PWUD and harm reduction organizations. These participants noted the difficulty PWUD face in navigating the healthcare and legal systems which are not built to serve PWUD’s unique needs and how these struggles create a strong bond within their communities. A large majority of PWUD participants reflected on their community’s resilience in caring for someone experiencing an overdose. For example, one PWUD participant shared: “The police and EMS…they’re usually not the first ones there. It’s the people who, like, are in the household or in the community that are using with them that…are the ones who do the rescues” (125, PWUD). A harm reductionist highlighted the strength and resilience inherent in the drug using community: “PWUD have long ensured the health and safety of their own community. Mostly out of necessity but also out of choice and love. They make sure their community has what it needs to stay safe and alive without judgment and with dignity” (120, HR).

##### Harm reduction organizations facilitate better care and resources for PWUD

Harm reduction organizations are strong allies and advocates for PWUD in Texas. As one harm reductionist shared: “Texas is one of the most hostile states in terms of harm reduction work. Despite this, there are many harm reduction groups all over Texas who are doing amazing work, as well as a movement to unionize PWUD which is supported and fostered by harm reductionists” (140, HR).

Harm reductionist participants described how their passion and commitment to serving PWUD was exemplified in their response during the COVID-19 pandemic: “Harm reduction teams were in the streets doing the work during the worst times of COVID which speaks to the passion and dedication of these groups. With so many services closed and nearly impossible to access, harm reductionists understood that the needs of this increasingly vulnerable population continued to grow and we had to act, fast. Many people who use substances do not trust in the ‘system’ for a multitude of reasons” (179, HR).

#### Emergent themes pertaining to policy, state-level legislation, and funding structure

Participants reported several challenges related to implementing evidence-based harm reduction strategies due to macro-level issues such as existing policies and structure of funding sources. CAB members unanimously agreed that policy advocacy efforts should focus on strengthening the Good Samaritan Law to better protect vulnerable populations, modifying the existing drug paraphernalia laws to decriminalize fentanyl testing strips and syringe exchange programs, and collecting better data to inform action. One CAB member highlighted these goals: “We need to focus on a strong Good Sam law, decriminalization of syringes and fentanyl testing strips… We need broad destigmatization efforts across domains and sensible data-driven policy and laws” (El Paso region, Male). Perspectives related to specific policies in Texas are described below.

##### Good Samaritan law perspectives

It is important to note that these data were collected prior to Texas adopting H.B. No 1694, a partial Good Samaritan Law, in September 2021. The results described below should be interpreted within this historical context. We asked our CABs to provide insight into the current Texas Good Samaritan Law to address this historical gap in our data and have outlined their perspectives in Sect. 2.1.3.

##### Good Samaritan law directly influences PWUD willingness to call 911 for overdose incidents

Participants indicated that Texas’ lack of a comprehensive Good Samaritan Law, which provides legal immunity for those calling in a drug overdose, propagates the incrimination of PWUD. Specifically, participants expressed concerns related to a bystander calling 911 and experiencing legal repercussions as a result of drugs being present on the scene. For example, one harm reductionist shared: “Texas doesn’t have a Good Samaritan law that would protect a person legally. So, let’s say I have, you know, a pound of heroin in my house—and you’re at my house and you fall out from an overdose and I call the police. They will definitely be taking me to prison” (102, HR). Another concern voiced was related to a bystander taking a person experiencing an overdose to the hospital and experiencing legal repercussions. A PWUD participant shared: “He was telling me that there is no Good Samaritan law, and that he knows somebody who actually is in prison right now, because they took their spouse to the hospital who was overdosing, and so they ended up going to prison because they died” (129, PWUD).

##### Community advisory board perspectives on the Texas Good Samaritan Law effective September 2021

Harm reductionists expressed encouragement that Texas passed a partial Good Samaritan Law 2021 (H.B. No 1694) allowing bystanders who see someone experiencing an overdose to call emergency services with protection from prosecution (Tex).; however, they noted concerns with the language of the Bill, the lack of dissemination of the Bill among PWUD, and its limited protections for those most at-risk (e.g. people with a criminal record). One CAB member noted: “I don’t think a lot of people even know about that Good Sam law—and it’s not that ‘good’ anyway. It’s so confusing and convoluted by design and difficult to figure out…much less during a medical emergency. It’s almost worse than having no law—because it’s really of no practical benefit, yet law makers can make the argument that ‘Well, we already have a Good Sam law in place’” (Austin region, Male).

#### Drug paraphernalia laws limit overdose prevention efforts

Participants described current drug paraphernalia laws in Texas as a barrier to PWUD efforts to stay safe and harm reductionists’ overdose prevention efforts in terms of what supplies they can distribute. This theme emerged within the context of syringe service exchange and fentanyl testing strips: "…syringe exchange is not legal in Texas, and that’s really um stopping us from meeting then what we need to do—the people that we need to meet and the demand that we need to meet. We are very—we’re-we’re not doing it, we’re just not—we’re not doin’ it.”” (116, HR).

#### Funding structure for harm reduction efforts

Harm reductionist participants described perceived challenges inherent within the federal structure of funding sources for harm reduction services including continued access to naloxone, need for increased state-funded methadone slots, and the structure of block grants. One harm reductionist described challenges related to naloxone access: “Funds are no longer available to provide free Narcan [naloxone] to people and many pharmacies will still not provide Narcan to people without a prescription and [it’s] prohibitively expensive for most people. We must increase funding for harm reduction programs in order to ensure Narcan is readily available for those who need it” (140, HR). This quote demonstrates the persistent challenges community organizations face in obtaining a steady supply of naloxone even in a state that has dedicated substantial resources to making it readily available through a public website. Notably, there was a period of time in 2021–2022 where that website was out of stock due to an unexpected increase in requests. Participants discussed limitations of grants specifically noting how this funding structure fails to provide long-term stability of harm reduction programs which results in premature program closure and increased work-related stress and burnout as staff may not feel confident in job security within harm reduction organizations.

### Emergent themes pertaining to the carceral system

#### Impact of law enforcement structure on harm reduction efforts

Themes related to the carceral system emerged across all participant groups. Fear of legal repercussions and police involvement emerged as a theme among both harm reductionists and PWUD participants. Specifically, PWUD described fear pertaining to law enforcement involvement in overdose response. Almost all PWUD participants indicated that the prospect of getting arrested dissuades them from calling 911 following an overdose. One PWUD participant described: “I really don’t want to have to deal with police…because who knows, they may want to, um, take a walk through the apartment and they see something, get in trouble…I don’t know why getting in trouble seems to be so much worse than, um, dying from an overdose…I really don’t like the thought of jail” (133, PWUD).

Participants also expressed concerns of legal repercussions for bystanders of an overdose incident. Another PWUD participant reported: “I’ve heard a lot of people get in trouble from—from sticking around. I mean, that’s why, you know, we already now don’t stick around…we’re not that dumb to stick around…and then you have to go to County [jail]” (159, PWUD). Further, fear of the police coming to the emergency department following an overdose incident was prevalent: “When cops come to the hospital, and if they search you…you can go to jail. You can go to jail for overdosing because they search you and find drugs on you…I feel like that’s crazy…It’s like they would be happier if we would just die I guess” (129, PWUD).

#### Limited access to medication for opioid use disorder in jail

Several PWUD participants indicated that a large part of their fear of law enforcement was centered around experiencing withdrawal in jail and not having access to treatment resources once there. One participant specified that “the fear of going to jail is you’re in there sick” (128, PWUD). Another PWUD clarified the challenges of curbing withdrawal symptoms in a jail setting: “I’m scared of going to jail, especially if I’m strung out…because gettin' drugs in frickin’ jail sucks…I mean, they give you Benadryl and Vistaril, which is nothing. It literally has no effect whatsoever to help with the withdrawal symptoms” (135, PWUD). Another participant indicated that oftentimes PWUD do not get any assistance once in the jail setting: “When we get arrested, we don’t get no help in County [jail]. We just get thrown in a cell, and that’s it. We don’t get no help with withdrawal or nothing like that. They just leave us there to rot” (159, PWUD).

### Emergent themes pertaining to the healthcare system

#### Abstinence-based model prevails over harm reduction philosophy

Harm reductionists described the abstinence-based model of care as the prevailing philosophy surrounding substance use prevention and treatment in Texas. Participants perceived this as a challenge when harm reduction organizations attempt to collaborate with healthcare providers or when clients want to enter treatment. One harm reductionist described their experiences: “I mean, we’re trying to collaborate with a bunch of different pieces. We’re really trying to look at harm reduction as, like, an integrated care, right? Um, medical is difficult… getting medical services where we need them has proven to be difficult so far” (179, HR). Another participant noted, “Looking at abstinence-based-model programs… they don’t even wanna have a conversation about Narcan [naloxone]. They don’t even wanna give people Narcan when they leave treatment… that stigma is still there” (109, Harm Reductionist).

#### Insufficient and uncoordinated care continuum following an overdose incident

A key barrier noted within the healthcare and emergency management systems is the failure to provide appropriate referrals to addiction treatment or other services following an overdose treated in an emergency department. An emergency responder described the following: “A good example is we get called to them because it’s an emergency. Okay, well, we’re not gonna really do much. We’re gonna send [them] to the ER. Well, the ER is not gonna really do much. They’re gonna stabilize them and send 'em home” (174, EMS). A harm reductionist with lived experience added, “I was never provided with any resources…a referral to, uh, MAT [Medication-Assisted Treatment]…I was never provided with any of that…I would wake up in a room full of people that are like, ‘God, this dude’s in here again with this overdose, man…when is he gonna finally have enough?’” (110, HR).

#### PWUD experience drug use-related stigma from healthcare providers

Participants described how PWUD are stigmatized by healthcare providers, particularly in emergency department settings. One emergency responder described an overdose call after they see the patient at the scene, “Then you take them to the hospital. The hospital staff is rude, and you know, very condescending to the person—to the patient” (146, HR). A PWUD participant shared their experience: “Like I had a rake stuck in my foot for eight hours, and the doctor came in there…and they said, ‘Well maybe if you didn’t use drugs, we’d be able to give you something for your pain’” (125, PWUD). One participant highlighted the role that harm reduction organizations may assume as healthcare providers: “Harm reductionists may be the only ‘[healthcare] provider’ this population engages with which gives us the opportunity and responsibility to promote trust building in services again by ensuring we are consistent and operate with integrity” (179, HR).

### Emergent themes pertaining to the harm reduction infrastructure

#### Limited and unstable funding structures create staffing and data collection concerns

Most harm reductionist participants highlighted the importance of collecting data on their clients as data demonstrates the impact of their work in the community and facilitates applying for other sources of funding. However, participants also indicated that there is often insufficient guidance from funding sources and insufficient funding for staff to support strong data collection and record keeping within the organization. As one harm reductionist poignantly stated: “the person who’s in charge of the data reporting is also…our only HIV tester…there’s too much on her plate” (138, HR). Another harm reductionist indicated they were using a mobile application to track overdose data; however, following a change in funding source this mobile application was not supported at the organization level: “…once some funding shifts happened, it was no longer being used” (106, HR). Data collection during outreach also proves to be challenging: “The chaos that we see and manage…data collection is usually one of the things that falls to a lower priority…we have three outreach staff; one of them started last week…barriers [to collecting data] are just, like, all the other shit” (105, HR).

#### Need to improve equity in harm reduction service provision

Similar to PWUD, harm reductionists in Texas manage their organization operations in an increasingly difficult landscape. Many harm reductionists indicated that while their services positively impact their clients, many people who need services go unserved, emphasizing the need to increase access to services among women, people of color, LGBTQ populations, sex workers, and rural areas. One harm reductionist stated, “Women are not being reached. Um, black community is not being reached. Hispanics are not being reached. Uh, just poorer communities…So yeah, there’s a lot of people that we’re not reaching out to…or that don’t just seek out services…I feel like younger people, uh you know, teenagers…they’re not comin’ out” (116, HR). Another participant noted, "The lack of resources out in rural areas, um, and the native population. I think those are the two biggest ones that we have not been, um, successful in terms of reaching” (107, HR). Harm reductionists highlighted challenges for engaging outreach with some populations experiencing homelessness who are “hidden”: “They don’t want to be seen…you got people that stay in the woods, but you got people that stay even deeper in the woods, for an example. Those are the ones that, like, yeah, those are the ones, like how do you find them?…It’s mostly like the veterans. Um, yeah. A lot of trauma. PTSD and they keep away from a lot. Those are the ones to me that doesn’t wanna be messed with, contacted, anything like that” (104, HR).

## Discussion

This study used a CBPR approach to understand perspectives on harm reduction practices in Texas. Our study is the first to investigate strengths and challenges to harm reduction implementation among an array of stakeholders in Texas including PWUD, harm reduction workers, and first responders. Emergent themes highlighted strengths among PWUD and harm reduction organizations that have been grounded in a mutual experience of stigma and discrimination. Specifically, community resiliency and perseverance to protect each other through use of harm reduction practices emerged as a central theme. Harm reduction organizations have established a statewide collaborative network based in a person-centered approach to advocate for drug users’ rights and safety.

Study findings highlight how existing state policies may exacerbate the drug overdose crisis by impeding what harm reduction services can be provided and by whom. Unquestioningly, harm reduction services (e.g., syringe exchange, overdose education and naloxone distribution) and materials (e.g., fentanyl test strips, safe smoking kits) successfully reduce HIV and HCV transmission, reduce overdose death rates, reduces harmful drug use with safer strategies, and also facilitate entry into substance use disorder treatment [27–29]. There is immense need for evidence-based drug policies that support harm reduction practice and programs. PWUD have unique expertise to contribute towards the development of community-based harm reduction initiatives.; consequently, equitable compensation and decision-making power should be given to PWUD for their subject matter expertise.

PWUD described experiencing a host of barriers when interacting with the carceral and healthcare systems. Most salient across both systems included the sustained dominance of an abstinence-based philosophy pertaining to substance use disorders. Specific to the carceral system, participants noted a lack of access to medications for opioid use disorder and significant fear of police involvement following a person experiencing an overdose. Participants reported stigmatizing, dehumanizing, and discriminatory experiences that deterred them from interacting with either system at all, a barrier common to PWUD in the United States [30–32]. Our findings corroborate existing literature suggesting a continued need for bias and anti-stigma training for providers that serve PWUD, especially emergency responders and emergency department personnel [33, 34]. Further, participants noted a severe lack of coordination of overdose prevention and treatment services following discharge from the emergency department and community re-entry from the jail system.

Study findings demonstrated a need to improve the infrastructure for harm reduction organizations to operate within communities. Participants noted that limited and unstable funding sources create instability within harm reduction organizations. Further, better data collection and aggregation methods are needed to inform data-driven response efforts and promote equity in harm reduction service provision within the community. Staff and volunteers in harm reduction organizations experience significant work-related stressors including repeated exposure to clients’ experiencing an overdose or other vicarious trauma, financial insecurity, and lack of respect and recognition for their work as a harm reductionist [35]. All of which may directly facilitate burnout and staff turnover in harm reduction organizations. As such, mental health supports and trauma-informed interventions may improve organizational stability and operations within harm reduction organizations.

## Study limitations

Findings from this study should be considered in light of the following limitations. First, this is a secondary analysis of a larger qualitative study investigating overdose reporting in Texas. We did not directly probe for data related to policy and legislation perspectives on harm reduction. Similarly, we also did not directly ask about perceived strengths associated with harm reduction efforts in Texas. Future studies should explore these topics until saturation is reached. Second, we used convenience sampling for study recruitment which may have contributed to more consensus in study results relative to other sampling methodologies. Finally, this study was conducted among four diverse Texas counties. Although the geographic diversity of the sample is a strength, data may not be generalized to other areas of Texas such as the Rio Grande Valley, the Panhandle, East Texas, and tribal communities.

## Conclusions

Texas has demonstrated strong progress in reducing harm related to opioid use in recent years. Federal funding has supported state-level efforts to increase access to naloxone and treatment for opioid use disorder. Further, Texas has taken steps to improve policy related to harm reduction such as implementing a Naloxone Access Law that protects prescribers and dispensers from criminal and civil liability for distributing naloxone and a partial Good Samaritan Law that provides limited protection to bystanders contacting emergency services during an overdose. Taken together, this study provides data supporting avenues for enhancing harm reduction philosophy and practice across Texas including policy advocacy, improved funding support dedicated to harm reduction efforts, disseminating knowledge and reducing misinformation about harm reduction philosophy across the healthcare and carceral systems, and elevating the voice of PWUD to advance safer use initiatives and build trust.

## Data Availability

The data that support the findings of this study are available on request from the corresponding author, KC. The data are not publicly available due to their containing information that could compromise the privacy of research participants.

## Abbreviations

PWUD: People who use drugs
CAB: Community Advisory Board
CBPR: Community-based Participatory Research
HRW: Harm Reduction Worker
